# The Negative Effects of Feces-Associated Microorganisms on the Fitness of the Stored Product Mite *Tyrophagus putrescentiae*

**DOI:** 10.3389/fmicb.2022.756286

**Published:** 2022-03-10

**Authors:** Stefan J. Green, Marta Nesvorna, Jan Hubert

**Affiliations:** ^1^Genomics and Microbiome Core Facility, Rush University, Chicago, IL, United States; ^2^Crop Research Institute, Prague, Czechia; ^3^Faculty of Agrobiology, Food and Natural Resources, Czech University of Life Sciences Prague, Prague, Czechia

**Keywords:** mite, feeding, microorganisms, feces, transmission, yeasts

## Abstract

Feces have been suggested as a major source of microorganisms for recolonization of the gut of stored product mites via coprophagy. The mites can host microorganisms that decrease their fitness, but their transmission is not known. To address the role of fecal microbiota on mite fitness, we performed an experimental study in which the surfaces of mite (*Tyrophagus putrescentiae*) eggs were sterilized. Mites eggs (15 per experimental box) were then hatched and grown on feedstock with and without feces. These experiments were conducted with four distinct *T. putrescentiae* populations (5L, 5K, 5N, and 5P), and mite population density after 21 day of cultivation was used to assess mite fitness and the impact of fecal microbiota on fitness. Population density was not affected by the presence of feces in two of the cultures (5L and 5K), while significant effects of feces were observed in the other cultures (5N and 5P). Mite culture microbial communities were analyzed using cultivation-independent next-generation amplicon sequencing of microbial 16S and 18S ribosomal RNA (rRNA) genes in the fitness influenced populations (5N and 5P). Several microbial taxa were associated with fecal treatments and reduced mite fitness, including *Staphylococcus* and *Bartonella*-like bacteria, and the fungal genera *Yamadazyma*, *Candida*, and *Aspergillus*. Although coprophagy is the transmission route mites used to obtain beneficial gut bacteria such as *Bartonella*-like organisms, the results of this study demonstrate that fecal-associated microorganisms can have negative effects on some populations of *T. putrescentiae* fitness, and this may counteract the positive effects of gut symbiont acquisition.

## Introduction

Coprophagy, the eating of feces, is one of the mechanisms of beneficial microbe transmission in gregarious insect taxa (Nalepa et al., 2001; Onchuru et al., 2018). Coprophagy is a predominant route of symbiont transmission for insects belonging to the order Hemiptera and Blattodea (Salem et al., 2015a). That coprophagy mediates the host–microbial interactions has been demonstrated in a number of experiments/studies/insects (Engel and Moran, 2013; Onchuru et al., 2018; Jahnes et al., 2019; Hosokawa and Fukatsu, 2020). In one such study with germ-free and gnotobiotic cockroaches (*Periplaneta americana*) the former exhibited prolonged growth rates and gut tissue dysmorphias (Jahnes et al., 2019). Furthermore, gut-associated bacteria have been shown to mediate aggregation of the cockroach species *Blattella germanica* and aggregated cockroaches feed intensively on their own feces (Wada-Katsumata et al., 2015). Coprophagy facilitates vertical transmission when the feces are deposited alongside the eggs or offspring. Under these conditions, the offspring are inoculated with bacteria during feeding on parental feces (Onchuru et al., 2018). However, the transmission of mutualistic microbes across host generations entails a significant risk of co-transmission of pathogens or parasites. For example, Salem et al. (2015b) have shown transmission of symbiotic bacteria *Coriobacterium glomerans* and *Gordonibacter* sp. in the host insect *Dysdercus fasciatus*, and transmission of the trypanosomatid parasite *Leptomonas pyrrhocoris*.

Stored product mites have gregarious feeding patterns (Levinson et al., 1991a,b) and feeding occurs in massive aggregations in infested food (Garcia, 2004; Mueller et al., 2006) and homes (Baker and Swan, 2013). These mites also are well documented carriers of microorganisms; microorganisms either stick to their bodies and setae or are carried inside the organism in the digestive tract and can be transferred via feces (Griffiths et al., 1959; Hay et al., 1992). Mites of the species *Acarus siro* have been shown to host filamentous bacteria in the gut diverticula (Sobotnik et al., 2008), suggesting a symbiotic relationship based on their localization. In addition to bacteria, microanatomic observations have shown that fungal spores persist during passage in the mite gut (Smrz and Catska, 1989), and manipulative experiments have confirmed that fungi can colonize new food commodities via transmission by mites (Franzolin et al., 1999) and mites are able to develop on wide spectrum of fungal species (da Silva et al., 2019).

We previously studied the role of fecal transmission of bacteria in the mite species *Tyrophagus putrescentiae* through an experimental study in which microorganisms isolated from feces were added into the mite diet (Hubert et al., 2018). Although in one culture no effect of microbial addition was observed, a second mite culture had increased fitness and increase in the relative abundance of *Bartonella*-like bacteria from 6 to 20% in their microbiomes (Hubert et al., 2018). Conversely, in a laboratory culture of *T. putrescentiae* containing *Bacillus cereus* growing on feces, mite dead bodies and exuvia, microbe additions into diet decreased mite fitness (Erban et al., 2016b). These studies indicate the potential for host fitness to be affected by fecal-associated microorganisms during rapid growth of mites, but more studies are needed to assess the importance of feces and fecal-associated microorganisms for mite growth.

In this study, we performed an experimental manipulation to examine mite growth in conditions with and without mite feces (cleaned and feces treatments). Surface-sterilized mite eggs were used to seed mite growth on a diet with or without amended mite feces, and the effect of treatment was compared. Mite fitness was measured as the population density in growth chambers after 21 days of cultivation from 15 mite eggs. We determined the changes in microbiomes in of mite cultures between selected populations (5N and 5P) in cleaned and feces treatments. We sampled and analyzed the microbiome from the whole chambers which includes the mites, their feces, remains of their bodies, and the diet.

## Materials and Methods

### Mites

Four populations of *T. putrescentiae* were used in this experiment: (i) 5L, a laboratory population collected by E. Zdarkova in a grain store in Bustehrad, Czechia in 1996; (ii) 5N, a canine-associated population collected in a meat-producing factory, St. Louis, MO, United States in 2007; 5K, from the Koppert-rearing facility, E. Baal in 2012, Netherlands; and 5P, from a laboratory strain, T. Phillips in 2014, Manhattan, Kansas, United States. The mites were kept in our rearing facility from the time of collection on stored product mite diet consisted of mixture of oat flakes, wheat germs, and Mauripan dried yeast extract (AB Mauri, Hampton, Peterborough, United Kingdom) (ratio 10:10:1 wt) (5L, 5K, 5P) or a dog food kernel diet (5N). A detailed protocol describing mite rearing has been published previously (Erban et al., 2016a; Hubert et al., 2018; Nesvorna et al., 2019). These mite populations have differing intracellular symbiont compositions. Culture 5L mites are colonized by *Cardinium*, while culture 5N and 5P mites are colonized by *Wolbachia.* Culture 5K mites are asymbiotic. *Bartonella*-like bacteria and *Bacillus* have been detected in feces from all four studied cultures (Erban et al., 2016a; Hubert et al., 2018; Nesvorna et al., 2019).

### Experimental Growth Chambers

Experimental mite growth chambers were modified slightly from those described previously (Hubert et al., 2001). Briefly, the chambers consisted of a lower microscope glass slide (76 mm × 26 mm), filter paper covering the slide (76 mm × 26 mm), a steel washer (30 mm diameter, 10 mm inner diameter), and a microscope glass slide on top of the washer. The glass slides were held onto the washer by metal crocodile clips. The filter paper was moistened by distilled water, and the chambers were stored at desiccators at 85% r.h. and 25 ± 0.5°C in darkness. Pasteurized food (70°C for 30 min) was added into each chamber.

### Isolation and Sterilization of Mite Eggs

To obtain eggs for the study, mites were raised on wheat germ sieved on 710 μm sieves (the sieved fraction was discarded). Five hundred adult mites per culture were transferred from rearing chambers into the new chambers by brush and incubated for 5 days. After 5 days of growth, the contents of the chambers were sieved using 100 μm sieves, and the sieved faction with the eggs and diet debris was transferred into 20 mL PBST (phosphate saline buffer with addition of Tween 20) and shaken overnight by 300 rpm at 25 ± 1°C. The PBST was then transferred into 50 mL falcon tubes after straining through mesh pluri-Strainers 40 μm (pluri-Select Life Science, Leipzig, Germany). The eggs were collected in the sieves, while the diet debris are sieved and discarded. The sieves with eggs were processed by washing in 5 mL of bleach (mixture of sodium hypochlorite 4.7%, sodium hydroxide <1%, SAVO original, Unilever, Prague, Czechia). After surface sterilization, the eggs and mesh were washed again (3×) in 5 mL of PBST. Finally, the eggs were removed from strainer by sterile brush and placed into experimental chambers using a dissection microscope.

### Isolation of Mite Feces

Strips of filter paper 76 mm × 25 mm were added into two-month-old mite cultures in tissue cultured flasks (catalog number 3103-025, Iwaki, Asahi Techno Glass Corporation, Chiyoda City, Tokyo, Japan). The papers were exposed to the cultures for 5 days and then removed. The paper was placed between two lower microscope slides and the mites were killed by crushing. The crushed filter paper was then introduced into each experimental chamber as a source of feces.

### Experimental Setup

The experiment consisted of two treatments: (i) clean filter paper in the experimental box with diet and 15 surface sterilized eggs and (ii) filter paper with mite feces in the box and 15 surface sterilized eggs. Both treatments contained pasteurized diets. The experiments were conducted with 12 replicates, and mites were maintained in controlled conditions and checked periodically by microscopy to observe mite hatching. After 21 days of cultivation, the experiment was terminated. The chamber was open and the whole chamber was submerged into 10 mL of ethanol using plastic jars (100 mL volume). Then the jars were shaken and the ethanol with the mites and, feces and diet debris were collected to 50 mL flacons. The falcon was shaken to obtain the homogenous distribution of mites in the mixture and 3 × 1 mL of ethanol was used to count the number of mites in the sample. The rest of the ethanol mixture was used for DNA isolation (see Supplementary Figure 1). Mite population growth was used as the fitness indicator (Matsumoto, 1965).

### Single Mite DNA Isolation

To verify the cleaning process, we selected and analyzed a single mite with a quantitative PCR (qPCR) approach. The experiment was conducted as described above, but using 50 eggs per experimental chamber, and with 10 replicates per treatment with the 5P culture only. After growth, individual mites were selected and crushed by a pipette tip and placed in a sterile PCR tube. Mites were visually inspected under a dissection microscope to confirm rupture of their cuticle. Subsequently, 3 μL of Proteinase K (20 mg/mL) and 50 μL Chelex (5%) (cat No C7901; Sigma-Aldrich, Saint Louis, MO, United States) were added. Tubes were incubated for 4 h at 56°C followed by 30 min at 100°C. Tubes were centrifuged at 3,000 *g* and the supernatant was used for PCR amplification. Because the dominant members of *T. putrescentiae* community are intracellular symbiont *Wolbachia* and gut symbiont *Bartonella*-like, we focused on their quantification by qPCR (Hubert et al., 2018) as suitable markers for feces transmitted (*Bartonella*-like) and eggs transmitted (*Wolbachia*) bacteria. Absolute quantification of *Bartonella*-like and *Wolbachia* taxa using qPCR targeting 16S RNA genes was performed as described previously (Kopecky et al., 2014; Nesvorna et al., 2019). Bacterial abundance was normalized to a per-mite basis.

### Microbial Community Characterization of Experimental Chambers

Mite culture microbial communities were analyzed using next-generation amplicon sequencing of microbial 16S and 18S ribosomal RNA (rRNA) genes in the fitness influenced populations (5N and 5P). We focus on the microbiome in both mites and their environment; therefore we analyzed the chamber contents including mites, their feces and exuvia and diet debris.

The falcons with ethanol were centrifuged 5 min 16,000*g* and then the ethanol was removed. The pellets were resuspended in 1 mL of ethanol and transferred to the Eppendorf tube. The tubes were centrifuged 5 min for 16,000 *g* and the ethanol was removed. After decanting the ethanol from the falcon and allowing residual ethanol to evaporate, the pellets were resuspended in the extraction 200 μL of extraction buffer from a Wizard SV Genomic DNA Purification kit (Cat No. A2365, Promega, Madison, WI, United States) and homogenized using a plastic pestle. The homogenate was incubated with 20 μL of proteinase K (20 mg/mL) at 56°C for 1 h. The lysate was purified using the purification kit, according to the manufacturer’s instructions. DNA was eluted in 100 μL of elution buffer.

Absolute quantification of 16S and 18S genes abundance in the samples (chambers) was performed as described previously (Kopecky et al., 2014; Nesvorna et al., 2019), and microbial gene abundance was normalized to a per experimental chamber basis.

Genomic DNA or lysates were processed for NGS amplicon sequencing using a two-stage PCR protocol, as described previously (Naqib et al., 2018). Briefly, samples were amplified with primers targeting fragments of microbial 16S rRNA genes (CS1_515pF and CS2_806aR) and fragments of fungal 18S rRNA genes (CS1_FF390 and CS2_FR1) (e.g., Walters et al., 2016). All primers contained 5’ linkers (Fluidigm Access Array linker sequences), as described previously (e.g., Naqib et al., 2018). PCRs were performed using Takara Ex Taq DNA polymerase and master mix (cat. no. RR001A, Takara Bio, Saint-German-en-Laye, France); PCR cycling conditions were as follows: 95°C for 5 min, followed by 28 cycles of 95°C for 30 s, 55°C for 45 s, and 72°C for 30 s, and finally, 72°C for 7 min. Every reaction was run in triplicate. Negative control reactions were generated by using 1 μL of ddH2O as the template. PCR products were visualized by agarose gel electrophoresis; technical replicates were pooled. Subsequently, PCR amplicons were prepared for sequencing by incorporation of sample-specific barcodes and Illumina sequencing adapters with PCR amplification Fluidigm Access Array for Illumina primers. The Fluidigm PCR cycling conditions were as follows: 95°C for 5 min, followed by 8 cycles at 95°C for 30 s, 60°C for 45 s, and 72°C for 30 s, and termination at 72°C for 7 min. Barcoded amplicons were pooled and sequenced on Illumina MiniSeq (16S; 2 × 153 base sequencing) or MiSeq (18S; 2 × 250 base sequencing) instruments. Fluidigm primers were used to initiate sequencing reactions according to the Access Array for Illumina primers manual. Barcoding PCR and sequencing were performed at the Genome Research Core, Research Resources Center, University of Illinois (Chicago, IL, United States). Raw sequence data were submitted to the Sequence Read Archive (SRA) at the National Center for Biotechnology Information (NCBI) website under the accession PRJNA686509 (Supplementary Table 1).

Raw sequence data were processed using the software package MOTHUR (Schloss et al., 2009), which was part of the MiSeqSOP pipeline (Kozich et al., 2013) according to the recommended protocol, including chimera removal by chimera.vsearch (Rognes et al., 2016) and taxonomic identification using the MOTHUR-formatted version of Ribosomal Database Project (RDP) (Cole et al., 2014) for bacteria. Sequences classified as chloroplasts, mitochondria or archaea were discarded. Then, the sequences were de-replicated, renamed and processed using the software package UPARSE (Edgar, 2013) according to the V4 pipeline protocol. Operational taxonomic units (OTUs) were generated at 97% similarity (OTUs_97_) using the cluster_otus command. Taxonomy was assigned using the UPARSE-modified RDP package (Edgar, 2018a,b) and Silva128 for fungi (Quast et al., 2013). Representative sequences for OTUs were analyzed using the Basic Local Alignment Search Tool (BLAST) to identify the closest sequences in the GenBank database (Altschul et al., 1990) (Supplementary Table 2). Datasets were rarefied to 5,000 reads per sample (Supplementary Tables 3, 4) for calculation of alpha diversity indices (e.g., Gihring et al., 2012).

### Statistical Analyses

Statistical analyses were performed in the software packages PAST (Hammer et al., 2001) and R 3.6.4 (R Development Core Team, 2017). Final mite densities were compared analyzed by Mann–Whitney test. Microbial community structure profiles were analyzed using the vegan package 2.5.7 in R (Oksanen, 2017). Independent variables (treatment, mite population, and mite density) that may affect microbial community profiles were analyzed by permutational multivariate analysis of variance (Anderson, 2001) using 10,000 permutations and Bray–Curtis matrices were used. The sample distribution was visualized by distance-based redundancy analyses (dbRDA) (Legendre and Anderson, 1999).

## Results

**FIGURE 1:**
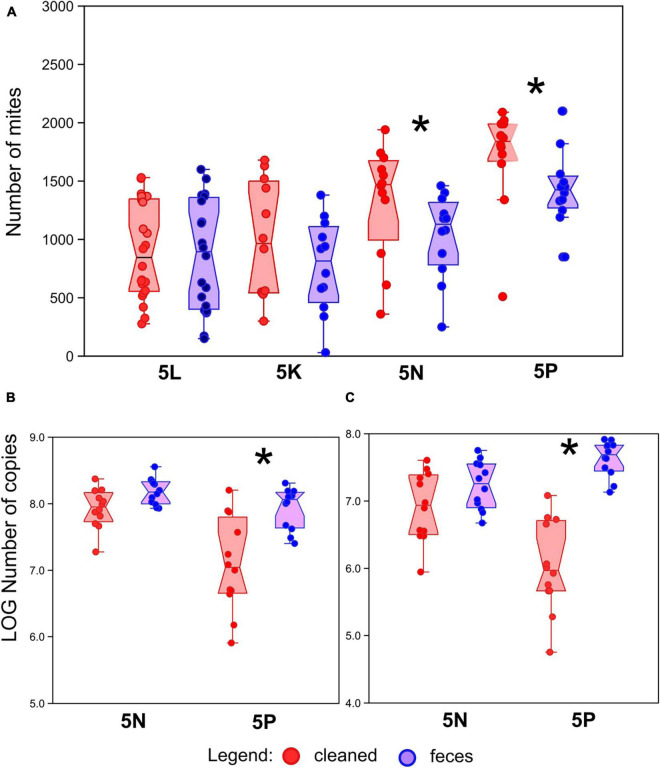
Effect of treatment on mite, bacterial and fungal abundance. **(A)** Number of mites in growth chambers after 21 days of growth with or without amendment of feces across four different mite populations (5L, 5K, 5N, and 5P). **(B)** Bacterial abundance, as measured by qPCR of 16S rRNA genes, in mite rearing chambers. **(C)** Fungal abundance, as measured by qPCR of 18S rRNA genes, in mite rearing chambers. Red symbols represent treatments containing surface-sterilized mite eggs and no mite feces. Blue symbols represent treatments containing surface-sterilized mite eggs and added mite feces. Significant differences (Mann–Whitney U-test, *P* < 0.05) are indicated by an asterix.

The final population density of mites was compared between treatments for each of the four populations (5L, 5K, 5N, and 5P; Figure 1A). No significant differences between treatment were observed for mite populations 5L and 5K (Mann–Whitney: z_(1_,_22)_ = 0.352, *P* = 0.724 and z_(1_,_22)_ = 0.81, *P* = 0.420, respectively). Conversely, significantly higher mite density was observed in the “cleaned” treatments for mite populations 5N and 5P (Mann–Whitney: z_(1_,_22)_ = 2.13, *P* = 0.031 and z_(1_,_22)_ = 2.20, *P* = 0.022, respectively). The population density was approximately 25% lower in fecal treatments than cleaned treatments for both 5P and 5N populations.

**FIGURE 2:**
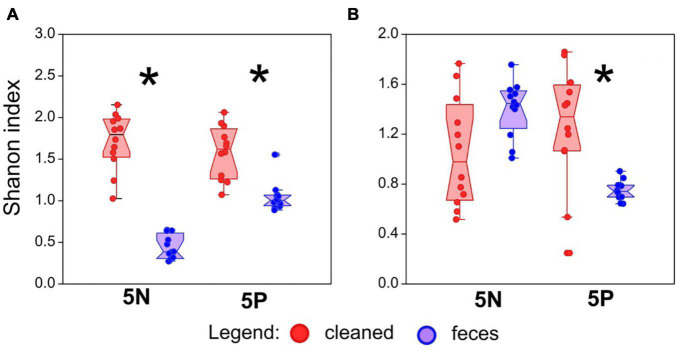
Effect of treatment on bacterial and fungal alpha diversity in mite rearing chambers. **(A)** Bacterial alpha diversity, as measured by Shannon index from 16S rRNA gene amplicon sequencing, in mite rearing chambers. **(B)** Fungal alpha diversity, as measured by Shannon index from 18S rRNA gene amplicon sequencing, in mite rearing chambers. Alpha diversity indices were calculated on rarefied datasets (5,000 sequences/sample). Red symbols represent treatments containing surface-sterilized mite eggs and no mite feces. Blue symbols represent treatments containing surface-sterilized mite eggs and added mite feces. Significant differences (Mann–Whitney U-test, *P* < 0.05) are indicated by an asterix.

**FIGURE 3:**
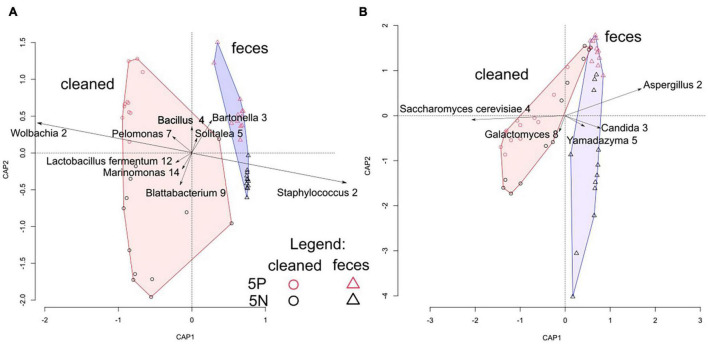
Correlation biplot of distance-based redundancy analyses of *Tyrophagus putrescentiae* growth chamber microbiomes. **(A)** dbRDA analysis of bacterial microbial communities assessed through 16S rRNA gene amplicon sequencing. **(B)** dbRDA analysis of fungal microbial communities assessed through 18S rRNA gene amplicon sequencing. Samples are color coded by culture (red = 5P; black = 5N) and by treatment (circle = cleaned treatment; triangle = feces treatment). Symbols represent individual samples, plotted in two dimensions, vectors represent OTUs, and convex hulls indicate cleaned and feces treated samples.

**FIGURE 4:**
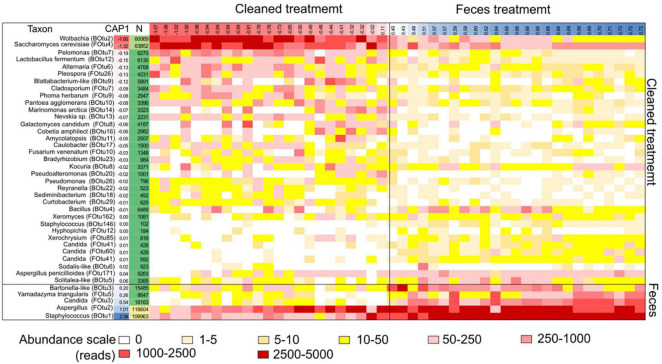
Heatmap of microbial taxa (OTU) correlated with mite fitness in 5N and 5P mite populations. Each row represents a specific microbial taxon and each column represents a specific growth chamber. The heatmap color scale is normalized to 5,000 sequences/sample. The samples are organized from left to right according to the CAP1 coordinates from the “total” dbRDA model for both bacterial and fungal OTUs for each sample (Table 1). The coordinates of the samples and OTUs are according to the first axes in dbRDA model. OTUs with fewer than 100 total reads are not shown.

To explain the decrease of the mite fitness in the feces-amended diets for 5N and 5P cultures, the microbiome of these cultures was analyzed using qPCR targeting bacteria and fungi (Figures 1B,C), and through 16S and 18S rRNA gene amplicon sequencing. Altogether we obtained 218 × 10^3^ bacterial reads (mean 4.5 × 10^3^) and 200 × 10^3^ fungal reads (mean 4.5 × 10^3^) (se Supplementary Figure 2 for rarefaction). The feces treated microbiomes showed the following features: (i) higher total microbial density (Figures 1B,C), significant in 5P, (ii) significantly lower Shannon diversity index (Figure 2) except for fungi in 5N, and (iii) significantly different microbial profiles (Figures 3, 4). Bacterial and fungal gene abundance was quantified using qPCR using universal 16S and 18S rRNA gene primers. Although the 18S rRNA gene qPCR assays can also target mites and other eukaryotic organisms, we have assumed that the 18S rRNA gene qPCR assay is primarily amplifying fungal sequences as mites were removed from the samples before extraction. Bacterial 16S rRNA gene abundances were significantly higher in feces treatment samples than in cleaned treatment samples 16S for 5P (Mann–Whitney: z_(1_,_21)_ = 2.92, *P* = 0.004; z_(1_,_21)_ = 4.13, *P* < 0.001), 16S for 5N (Mann–Whitney: z_(1_,_22)_ = 2.06, *P* = 0.039). Average 16S rRNA gene abundances were approximately 5- and 10-fold higher in the feces treatments in 5N and 5P respectively. 18S rRNA gene abundances were higher in feces treatment samples than in cleaned treatment samples (Figure 1C). This was significant for the 5P culture (Mann–Whitney: z_(1_,_21)_ = 4.128, *P* < 0.001). Average 18S rRNA gene abundance was nearly 80-fold higher in the feces treatments in 5P mites. For 5N culture, the differences in 18S rRNA gene copies were trending toward significance (Mann–Whitney: z_(1_,_21)_ = 1.877, *P* = 0.060), and average 18S rRNA gene abundance was approximately 7-fold higher in the feces treatment relative to the cleaned treatment.

**TABLE 1:** The comparison of the effect of the observed factor to microbiome of *Tyrophagus putrescentiae* profiles using ANOSIM and dbRDA.

Model	Factor	ANOSIM		dbRDA				
		*F*	*P*	*F*	*P*	*R* ^2^	CAP1	CAP2
Bacteria	Treatment	**103.93**	**0.001**	**92.05**	**0.001**	0.66	0.925	0.075
	Culture	**11.68**	**0.001**	**10.49**	**0.001**			
	Mite density	0.87	0.385	0.99	0.0336			
Fungi	Treatment	**43.05**	**0.001**	**37.63**	**0.001**	0.42	0.93	0.07
	Culture	**4.092**	**0.015**	**4.483**	**0.022**			
	Mite density	0.7145	0.477	0.69	0.48			
All	Treatment	**82.61**	**0.001**	**50.85**	**0.001**	0.56	0.932	0.061
	Culture	**8.17**	**0.001**	**5.43**	**0.006**			
	Mite density	0.705	0.471	0.568	0.626			

*The tested data set were for bacterial and fungal microbiome analyzed neither separately nor altogether. The treatment included the feces treatment of diet and clean diet inside the experimental rearing chambers. The culture means two cultures 5N and 5P. The significant differences are indicated by bold.*

Average bacterial alpha diversity, as assessed using the Shannon index, was approximately 5- and 2-fold lower in feces treatment mites than in control mites for 5N mites and 5P mites, respectively (Mann–Whitney: z_(1_,_21)_ = 4.128, *P* < 0.001) and 5P bacterial (Mann–Whitney: z_(1_,_21)_ = 3.84, *P* = 0.001). Average fungal alpha diversity (Shannon index) was approximately 2-fold higher in clean treatment mites than feces treatment mites for 5P mites (Mann–Whitney: z_(1_,_21)_ = 2.74, *P* = 0.006), while no trend was observed for 5N mites (Mann–Whitney: z_(1_,_21)_ = 1.92, *P* < 0.053).

Microbial community structure was significantly affected by treatment, and to a lesser extent by mite culture (Figure 3 and Table 1). The environmental variables “effects of mite density” or interactions between “mite culture and treatment” to microbial profiles were eliminated due to high collinearity. The analyses showed that the first dbRDA axes separated the cleaned (negative values) and feces treated samples (positive values) for both bacteria and fungi (Figure 3). Specific microbial taxa contributing to the differentiation of the overall microbial communities are indicated in Figure 4. The feces associated taxa had x coordinates ranging from 0.20 to 2.36 in dbRDA model, including the bacterial taxa *Staphylococcus* (BOtu1) and *Bartonella*-like (BOtu3) and the fungal taxa *Yamadazyma* (FOtu5), *Candida* (FOtu 3), and *Aspergillus* (FOtu2). The microbiota correlated with the cleaned treatment conditions included those with the highest negative coordinates on the x-axis. These included three bacterial taxa including *Wolbachia* (BOtu2), *Pelomonas* (BOtu7) and *Lactobacillus fermentum* (BOtu12) and one fungal taxon—*Saccharomyces cerevisiae* (FOtu4). Four taxa were negatively correlated with mite population growth, including *Staphyloccocus* (BOtu1), *Candida* (FOtu3) (Table 2), *Yamadazyma* (FOtu5), and *Kocuria* (BOtu8). Twelve taxa were positively correlated with mite population growth, including *S. cerevisiae* (FOtu4), *Wolbachia* (BOtu2), *Pelomonas* (BOtu7), *L. fermentum* (BOtu12), *Alternaria* (FOtu6), *Pleurospora* (FOtu26), *Cladosporium* (FOtu7), *Pantoea* (BOtu10), *Phoma* (FOTU9), *Nevskia* (BOtu13), *Caulobacter* (BOtu17), and *Malassezia* (FOtu94).

**TABLE 2:** Spearman correlation between numbers of reads in standardized microbiome dataset and numbers of mites *T. putrescentiae* in the experiment (feces treated or cleaned).

Otu_97_	Taxon	*N*	Spearman	*P*
FOtu2	*Aspergillus*	116,604	–0.21	0.142
BOtu1	***Staphylococcus***	106,963	–0.48	**0.0005**
FOtu4	*Saccharomyces cerevisiae*	63,852	0.32	**0.0256**
BOtu2	***Wolbachia***	60,069	0.51	**0.0002**
FOtu3	***Candida***	18,163	**−0.45**	**0.0011**
BOtu3	*Bartonella*-like	15,485	–0.15	0.3001
FOtu5	***Yamadazyma***	8,647	**−0.35**	**0.0149**
BOtu4	*Bacillus*	6,489	0.01	0.9644
BOtu7	***Pelomonas***	6,270	**0.53**	**0.0001**
BOtu12	***Lactobacillus fermentum***	6,130	**0.32**	**0.0264**
BOtu9	*Blattabacterium-*like	5,891	–0.17	0.2486
FOtu171	*Aspergillus penicillioides*	5,263	–0.25	0.0861
FOtu6	***Alternaria***	4,768	**0.52**	**0.0001**
FOtu26	***Pleospora***	4,231	**0.48**	**0.0009**
FOtu8	*Galactomyces candidum*	4,187	–0.25	0.0844
FOtu7	***Cladosporium***	3,484	**0.46**	**0.0011**
BOtu10	***Pantoea***	3,396	**0.47**	**0.0011**
BOtu8	***Kocuria***	3,371	**-0.32**	**0.0281**
BOtu5	*Solitalea*-like	3,369	–0.05	0.7176
BOtu14	*Marinomonas-*like	3,323	–0.18	0.23
BOtu16	*Cobetia*	2,982	–0.18	0.2079
FOtu9	***Phoma***	2,947	**0.48**	**0.0008**
BOtu11	*Amycolatopsis*	2,507	0.11	0.4718
BOtu13	***Nevskia***	2,231	**0.50**	**0.0006**
BOtu17	***Caulobacter***	1,500	**0.44**	**0.0026**
FOtu10	*Fusarium*	1,348	0.27	0.0595
FOtu162	*Xeromyces*	1,081	–0.21	0.1653
BOtu20	*Pseudoalteromonas*	1,001	–0.06	0.6547

*The -significant correlations are indicated by bold, blue color indicates positive and red color indicates negative corrrelations. The taxon with total numbers of rads higher than 1,000 in standardized dataset are showed.*

**FIGURE 5:**
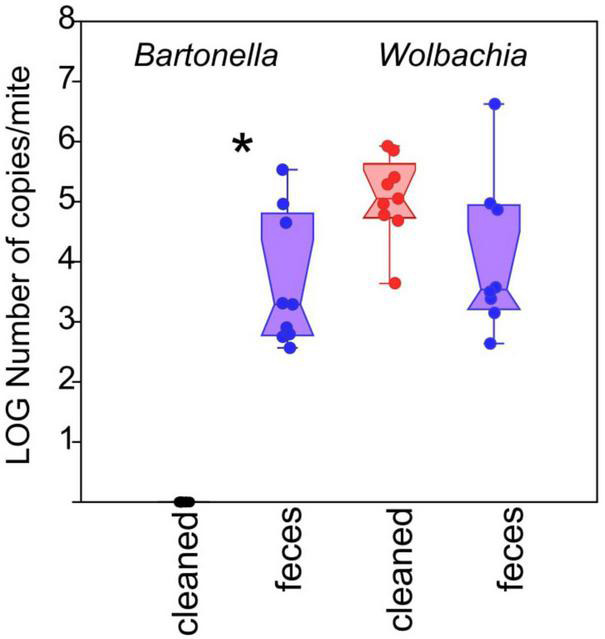
Effect of treatment on *Bartonella*-like and *Wolbachia* abundance in individual 5P culture mites. Absolute abundance of bacterial taxa, normalized to a per-mite basis, was measured using targeted qPCR. Red symbols represent treatments containing surface-sterilized mite eggs and no mite feces. Blue symbols represent treatments containing surface-sterilized mite eggs and added mite feces. Significant differences (Mann–Whitney U-test, *P* < 0.05) are indicated by an asterix.

We also assessed the effect of mite egg washing and treatment on the presence of *Bartonella*-like bacteria using qPCR in 5P mites. The cleaned mites did not contain any feces/gut-associated *Bartonella*-like bacteria, while feces treated mites contained approximately 10^3^ (median and interquartile range of 10^2^–10^4^) *Bartonella*-like copies per mite (Figure 5; Mann–Whitney: z_(1_,_16)_ = 3.775, *P* < 0.001). The abundances of the intracellular symbiont *Wolbachia* trended toward significance (Mann–Whitney: z_(1_,_16)_ = 1.876, *P* ≤ 0.061), and were higher in cleaned treatment mites (approximately 10^4^; median and interquartile range of 10^3^–10^5^) relative to feces treatment mites (approximately 10^5^; median and interquartile range of 10^4^–10^5^) (Figure 5).

## Discussion

Our study examined the role of coprophagy on mite growth fitness and on mite-associated microbial communities. Based on our previous experimental work examining bacterial addition to mite diet (Hubert et al., 2018), we expected that mites’ feces can serve as critical vectors for microbial transfer across generations and is beneficial to *T. putrescentiae*. Overall, however, we observed no positive benefits to feces amendment to mite diets. For two cultures (5L and 5K), we observed no significant effect on mite fitness, while for two others (5P and 5N) a decreased fitness was observed in the feces treatments. We, therefore, hypothesized that either global shifts in the mite-associated microbiome or specific fecal-associated microorganisms are responsible for the observed fitness decrease of the 5P and 5N mites.

Microbial community analyses identified significant profile changes between cleaned and feces treatment mites and enabled the identification of feces-associated taxa and taxa correlated with mite fitness. We focused on the analyzes of the whole microbiome from chambers which includes the mites, their feces and remains of their bodies and the diet. The taxa associated with the clean treatment mites are derived from internal symbionts *Wolbachia* (Werren, 1997), from the diets themselves [*S. cerevisiae*, *L. fermentum* (Klimov et al., 2019; Nesvorna et al., 2021)], or random taxa that have not been previously detected in mite diet studies (e.g., bacteria from the genera *Marimomonas*, *Nevskia*, and *Cobetia*). Although the cleaned treatments had lower bacterial and fungal abundances than fecal treatments, as measured by qPCR of 16S and 18S rRNA genes, bacterial and fungal loads were still high (e.g., >10^6^ copies/chamber for bacteria and >10^5^ copies/chamber for fungi).

The feces-associated taxa included *Staphylococcus* (BOtu1), which have been detected in high abundance in mite environments previously. The genus *Staphylococcu*s is ubiquitous and is frequently detected in mites, e.g., *Psoroptes ovis* (Hogg and Lehane, 1999; Oliveira et al., 2006) and *Demodex folliculorum* (Clifford and Fulk, 1990). *Staphylococcus* were present in most fecal microbiomes of various *A. siro*, *Carpoglyphus lactis* and *T. putrescentiae* cultures (Hubert et al., 2021). *Bartonella*-like (BOtu3) have been previously considered as being derived from the gut and feces of mites (e.g., Valerio et al., 2005; Hubert et al., 2021), and we confirm these prior findings here. For the fungal communities, the genera *Yamadazyma* and *Candida* were associated with the fecal treatments. Although Saccharomyces cerevisise was a compound of mite diet, the other yeasts (*Candida*, *Hyphopichia*, and *Malassezia*) have been previously identified in mite cultures and have been associated with house dust mite culture development and were associatiated with mite feces (Klimov et al., 2019; Molva et al., 2019; Nesvorna et al., 2021). *Yamadazyma* and have been elsewhere isolated from insects, also (Suh et al., 2008). Most critically, we observed negative correlations between mite density and specific microbial OTUs, including *Staphyloccocus* (BOtu1), *Candida* (FOtu3), *Yamadazyma* (FOtu5), and *Kocuria* (BOtu8). *Aspergillus* (FOtu2) was also observed to be associated with feces in support of prior studies demonstrating *Aspergillus* as associated with house dust mite cultures (Douglas and Hart, 1989). Previously, experiments with mites of the genus *Dermatophagoides* showed that while *Aspergillus penicillioides* decreased *Dermatophagoides* fitness, the second *Dermatophagoides* generation was unable to survive without *Aspergillus* in the mite diet (Hay et al., 1993). In this study, we used a different design, but the pasteurized diets were more suitable for mite fitness than feces treated.

While we observe specific microbial taxa associated with decreased mite fitness, we have previously observed positive effect of *Bartonella*-like bacteria in the feces on *T. putrescentiae* fitness (Hubert et al., 2018). *Bartonella*-like bacteria were present in the current study, but only in those chambers in which fecal material was amended. However, in the 5P mites, *Bartonella*-like bacteria were completely removed in the cleaned treatment, and these mites had the highest number of mites after 21 days of growth and lower average bacterial and fungal loads relative to the 5N mites. Thus, it is clear that *Bartonella*-like bacteria are not required for *T. putrescentiae* fitness. It is also possible that the positive presence of *Bartonella*-like bacteria cannot overcome the suppressive effects of the fecal-associated microorganisms that are correlated with decreased mite fitness. We have previously shown that some of the fecal treatment-associated microorganisms (i.e., *Staphylococcus*, *Candida*, and *Aspergillus*) are correlated to the mite waste deposit guanine (Nesvorna et al., 2021), and we hypothesize that these microbes decrease mite fitness either through the production of toxic metabolic wastes or through direct competition with the mites to nutrients, or both. In laboratory experiments, mite population growth has shown density-dependent patterns (Pekar and Hubert, 2008; Rybanska et al., 2016), which lead to decreased growth rates at higher densities. The pathogenic microflora has been suggested as partially responsible for such density-dependent growth inhibition. The negative correlation between mite density and specific feces-associated microbes suggested a regulation of mite population by these organisms in mite cultures. Such situation is probably not unique to mite cultures, but can occur in the infested commodities (e.g., cheeses, ham, dog food) when mite populations are of massive density (Aygun et al., 2007; Brazis et al., 2008; Canfield and Wrenn, 2010; Abbar et al., 2016). The presence or absence of pathogenic microflora may modulate the level of contamination of these stored commodities.

Prior studies using bleach surface cleaning of mite eggs showed that the eggs still contained viable surface associated microflora, due to surface structures (Kucerova and Stejskal, 2009; Hubert et al., 2021). For example, the washing of eggs of the striped shield bug (*Graphosoma lineatum*) in the ethanol did not eliminate the surface microbiota. Conversely, washing the eggs with 12% hypochlorite sodium for 14 min almost eliminated bacteria, but at the expense of a very low egg hatching rate (Karamipour et al., 2016). Eventually, additional time washing in 10% hypochlorite sodium provides for a feasible approach for bacterial elimination and egg survival (Karamipour et al., 2016). We have improved upon the prior surface sterilization methods by repeat washing in a lower concentration hypochlorite solution. qPCR results with taxon-specific primers showed that the surface associated *Bartonella*-like bacteria were completely removed, while the internal bacteria *Wolbachia* were still present. In addition, the eggs were viable and mite hatching followed bleach treatment. The next indirect measures of successful surface cleaning are: (i) the lower number of bacterial and fungal rRNA gene copies in cleaned treatments relative to feces treatments and (ii) the pronounced presence of diet-associated bacterial and fungal taxa (e.g., *S. cerevisiae* and *L. fermentum*) in the microbiome of cleaned mites. Both *S. cerevisiae* and *L. fermentum* have been previously shown to be replaced during mite culture growth (Klimov et al., 2019; Nesvorna et al., 2021). These data, together with the higher alpha diversity in cleaned treatment samples relative to feces treatment samples, indicate that the microbial communities that developed in the cleaned treatment samples are based on the environment (diet) and the intracellular community.

## Data Availability Statement

The datasets presented in this study can be found in online repositories. The names of the repository/repositories and accession number(s) can be found in the article/Supplementary Material.

## Author Contributions

JH and SG: scientific writing. SG: barcode sequencing. MN: qPCR and experiments. JH: bioinformatics analysis. All authors contributed to the article and approved the submitted version.

## Conflict of Interest

The authors declare that the research was conducted in the absence of any commercial or financial relationships that could be construed as a potential conflict of interest.

## Publisher’s Note

All claims expressed in this article are solely those of the authors and do not necessarily represent those of their affiliated organizations, or those of the publisher, the editors and the reviewers. Any product that may be evaluated in this article, or claim that may be made by its manufacturer, is not guaranteed or endorsed by the publisher.
