# Supplementary material for: The Negative Effects of Feces-Associated Microorganisms on the Fitness of the Stored Product Mite Tyrophagus putrescentiae
Source: Front Microbiol. 2022 Mar 10;13:756286. doi: 10.3389/fmicb.2022.756286 (PMC8961420; doi:10.3389/fmicb.2022.756286)
Supplement: Supplementary file 1 [file Data_Sheet_1.PDF]

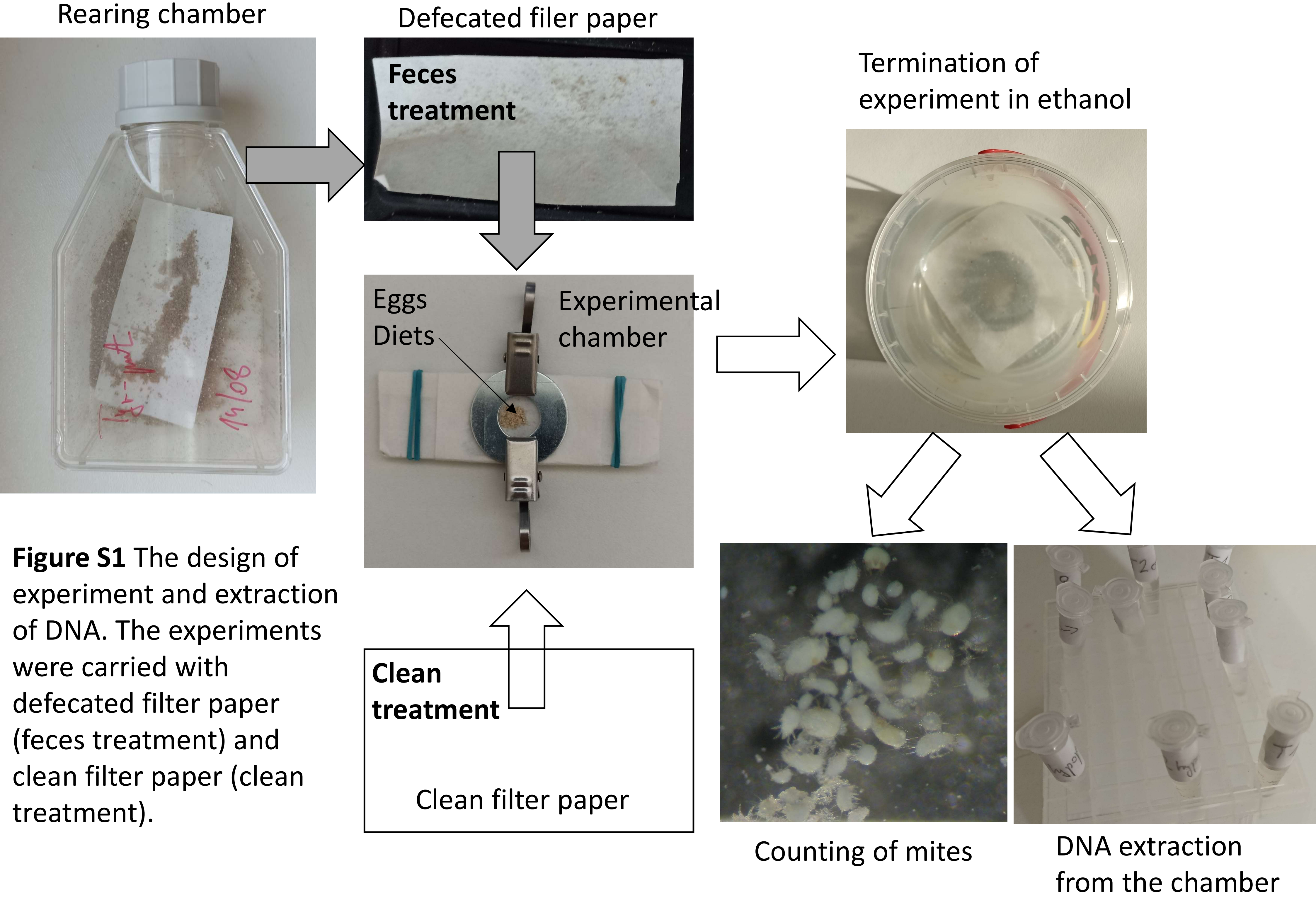

Figure S1 The design of experiment and extraction of DNA. The experiments were carried with defecated filter paper (feces treatment) and clean filter paper (clean treatment).
