# Supplementary material for: The Negative Effects of Feces-Associated Microorganisms on the Fitness of the Stored Product Mite Tyrophagus putrescentiae
Source: Front Microbiol. 2022 Mar 10;13:756286. doi: 10.3389/fmicb.2022.756286 (PMC8961420; doi:10.3389/fmicb.2022.756286)
Supplement: Supplementary file 2 [file Data_Sheet_2.PDF]

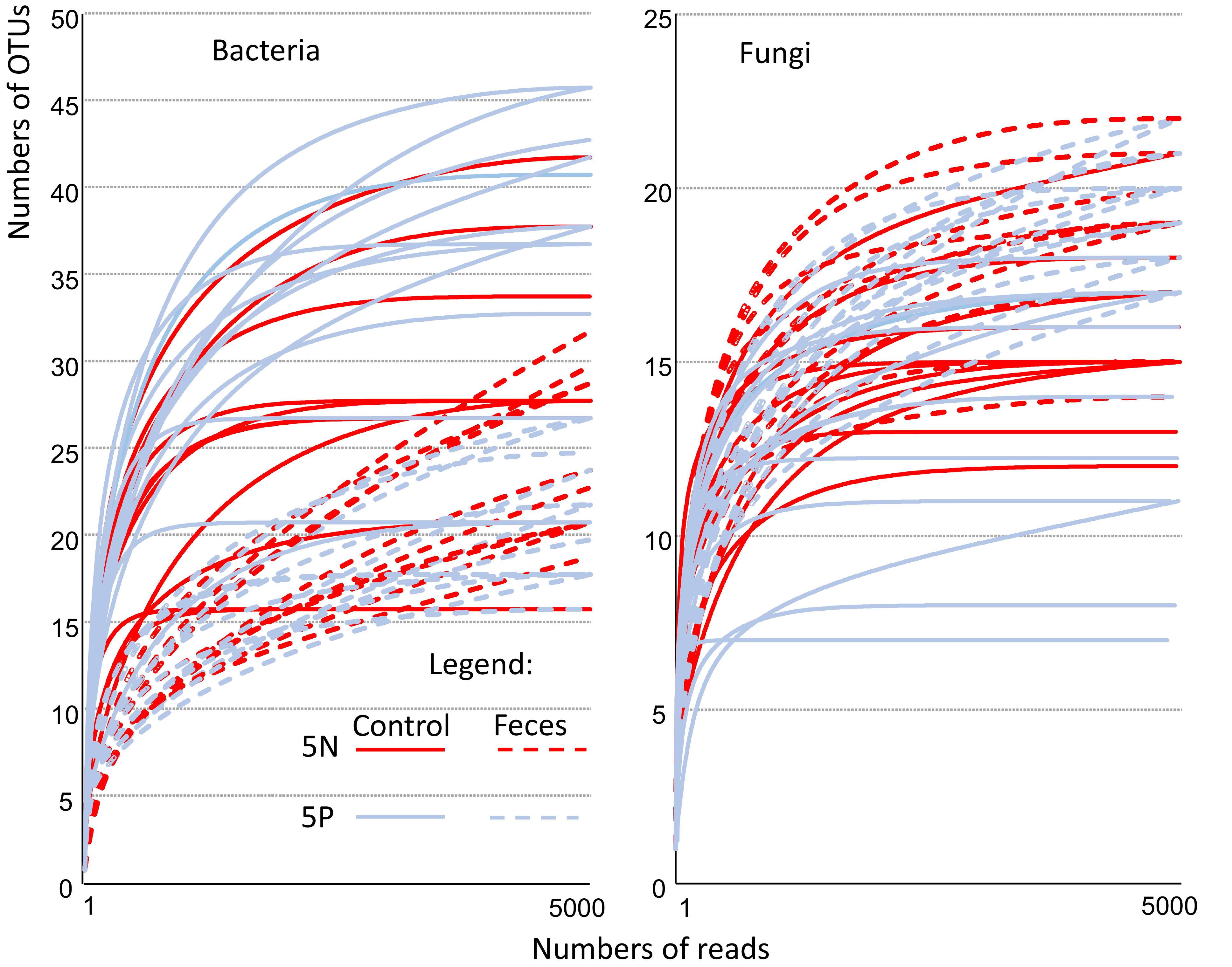

Figure S2. The rarefaction analysis of *Tyrophagus putrescentiae* microbiomes in the chambers contained feces treated and control diet.
